# Impact of a community-based asynchronous review clinic on appointment attendance delays across an eye hospital network in London, UK: an interrupted time series analysis

**DOI:** 10.1136/bmjopen-2025-098820

**Published:** 2025-07-15

**Authors:** Siyabonga Ndwandwe, Dun Jack Fu, Joy Adesanya, Juan Carlos Bazo‑Alvarez, Angus I G Ramsay, Naomi J Fulop, Josefine Magnusson, Steve Napier, Jocelyn Cammack, Helen Baker, Stephanie Kumpunen, Germán Andrés Alarcón Garavito, Holly Elphinstone, Grant Mills, Peter Scully, Anne Symons, Paul Webster, Jonathan Wilson, Peng Tee Khaw, Sobha Sivaprasad, Hari Jayaram, Paul J Foster, Caroline S Clarke, On behalf of the HERCULES Research Consortium

**Affiliations:** 1 Research Department of Primary Care and Population Health, University College London, London, UK; 2 National Institute for Health and Care Research (NIHR) Biomedical Research Centre (BRC) at Moorfields Eye Hospital NHS Foundation Trust and UCL Institute of Ophthalmology, London, UK; 3 Moorfields Eye Hospital NHS Foundation Trust, London, UK; 4 Escuela de Medicina, Universidad Cesar Vallejo, Trujillo, Peru; 5 Research Department of Behavioural Science and Health, University College London, London, UK; 6 Institute of Epidemiology and Health Care, University College London, London, UK; 7 University College London The Bartlett School of Sustainable Construction, London, UK; 8 University College London The Bartlett School of Architecture, London, UK; 9 Ubisense Ltd, Cambridge, UK

**Keywords:** Glaucoma, Diabetic retinopathy, Health Workforce, OPHTHALMOLOGY

## Abstract

**Abstract:**

**Objective:**

To assess the impact of opening a large community-based asynchronous review ophthalmic clinic on attendance delays among patients with stable chronic eye disease attending a London teaching eye hospital network.

**Design:**

Interrupted time-series analysis of routine electronic health records of appointment attendances.

**Setting:**

A large eye hospital network with facilities across London, UK, between June 2018 and April 2023.

**Participants:**

We analysed 69 257 attendances from 39 357 patients, with glaucoma and medical retina accounting for 62% (n=42 982) and 38% (n=26 275) of visits, respectively. Patients over 65 made up 54% (n=37 824) of attendances, while 53% (n=37 014) were from the more deprived half of the population, and 51% (n=35 048) were males.

**Intervention:**

An asynchronous review clinic opened in a shopping centre in London, in autumn 2021, following the COVID-19 lockdown in spring 2020.

**Main outcome measures:**

Average attendance delays (days), calculated as the difference between follow-up attendance date and the latest clinically appropriate date determined at the preceding attendance.

**Results:**

Pre-COVID-19, attendance delays for chronic eye disease monitoring were increasing by 0.9 days per week (95% CI, 0.8 to 0.9) on average, worsening to 2.0 days per week (95% CI, 2.0 to 2.0) after the first COVID-19 national lockdown, mid-March 2020. Opening the asynchronous review clinic increased appointment capacity, with delays decreasing on average by 8.1 days per week (95% CI, 8.1 to 8.2) shortly after opening. The rate of decrease slowed to 0.3 days per week (95% CI, 0.3 to 0.3) after 5 months. We found no significant differences in average attendance delays by age, gender or level of deprivation.

**Conclusion:**

The asynchronous review clinic significantly reduced attendance delays across the hospital network, addressing pre-existing backlog for stable chronic eye diseases. The reduction appeared to be maintained after the initial backlog had been cleared.

**Strengths and limitations of this study**

- The dataset included all patients who had appointments within the eye hospital network, thus covering a broad demographic range within London.
- We have used an interrupted time series analysis, a robust quasi-experimental design particularly suited for comparative evaluations where randomisation is not feasible.
- We did not have an external control group, which might limit comparison to national or regional trends.
- Incomplete and ambiguous ethnicity category labels limited equity-focused analyses. However, we included level of deprivation (IMD), mitigating some concerns about the equity impact of relocating services.
- Our analysis focused on glaucoma and medical retina patients only, potentially underestimating impact due to service expansion later on to other indications.

## Introduction

 England’s National Health Service (NHS) is under immense strain.^1^ Austerity measures, underinvestment in infrastructure, workforce shortages and rising demand for healthcare services have left the NHS struggling to keep pace, leading to significant appointment delays.^1^ Ophthalmology, the busiest outpatient specialty in England—accounted for approximately 8% of outpatient visits in 2022/2023^2^—is particularly impacted by these pressures. In March 2023, 628 502 people were awaiting ophthalmology appointments in England, with 27 260 of those having waited a year or more.^3^ Delayed follow-up appointments have had harmful impact on patient health outcomes, with previously ‘low-risk’ patients suffering severe vision loss.^4 5^ This limits their economic participation, costing the UK economy up to £7.4 billion annually in productivity loses.^6^

Both NHS England—which supports local health systems and providers to address healthcare inequalities while enhancing efficiency and care quality—and the Royal College of Ophthalmologists have recognised asynchronous review or virtual review clinics as an important service innovation that has the potential to improve service delivery.^7 8^ The service delivery model separates patient examination from clinician assessment, wherein the patient attends in-person routine monitoring tests performed by trained healthcare technicians. These tests are then reviewed by a clinician later to give a clinical diagnosis and management plan, which is reported back to the patient by letter. This conserves the limited cadre of specialist face-to-face attendancesfor more complex cases and those requiring urgent treatment.^9 10^

In September 2021, we initiated Project HERCULES (the Healthcare Exemplar for Recovery from COVID-19 Using Linear Examination Systems) and created an innovative, reconfigurable ‘laboratory of clinical efficiency’ in a shopping centre in London, UK (henceforth referred to as ‘intervention site’). Tests were performed by newly recruited, healthcare-naïve ophthalmic technicians who underwent 6–12 weeks of intensive training with continued support from senior clinicians. Their knowledge and skills were formally assessed 6 months after beginning their training. Tests at the intervention site were carried out according to the eye care facility network’s standard operating procedures and differed between disease categories, per guidelines. Test scans were uploaded to the hospital network’s secure online system and later reviewed remotely by specialist clinicians, who determined whether to discharge the patient, continue follow-ups through the asynchronous/virtual pathway or escalate to a face-to-face follow-up. The project significantly expanded the hospital network’s capacity, adding 38 500 appointment slots per year. Initially, services were for monitoring patients with stable glaucoma, age-related macular degeneration and diabetic retinopathy (henceforth broadly categorised as glaucoma and medical retina). This later expanded to include newly referred patients as well as those with keratoconus and cataract. The project is built on lessons from previously successful service innovations that this group has implemented, highlighting a potentially important strategy for easing NHS workforce and estate pressures.^11 12^

Previous research in ophthalmology has suggested that asynchronous review clinics are effective in managing stable patients given the typically slow disease progression and regularity of required reassessments,^11^ with more patients monitored via these technician-led clinics than at conventional clinics with clinical specialists on hand.^13 14^ However, the current literature provides limited evidence quantifying this service model’s effect on attendance delays. This work bridges this evidence gap by analysing appointment attendance trends before and after opening the asynchronous review clinic, taking the COVID-19 lockdown closures into account. The analysis used routine electronic health records of all eligible patients diagnosed with stable glaucoma and medical retinal conditions across a large eye hospital network in London and attended between 2018 and 2023.

## Methods

### Data sources

We retrieved 475 565 electronic attendance records for 96 088 glaucoma and medical retina patients who attended sites across the hospital network between June 2018 and April 2023. We excluded attendances where: patients were younger than 18 years (ineligible) or older than 100 years old at their first attendance (data quality issue); the site used a different electronic medical record system or had a major operational restructuring during the observed period (data quality issues); or the latest clinically appropriate date (LCAD) stated by clinician at the preceding attendance was longer than 18 months (medical retina) or 24 months (glaucoma) (data quality issues). We further excluded non-stable patients. For purposes of this work, we defined stable patients as those with an LCAD longer than 6 months from their previous attendance. Additionally, we excluded appointments taking place 4 or more weeks sooner than the stated LCAD. The former would indicate that the patient’s condition was not stable, while the latter would suggest an urgent visit might have occurred, that is, the attendance would not have been the scheduled regular monitoring visit. Patients diagnosed with both glaucoma and medical retina were retained in each analysis due to the distinct pathways for these conditions. Where patients had multiple visits per week, the initial attendance was retained while subsequent attendances were excluded, due to the weekly panel nature of the analysis.

### Outcome measures

Our primary outcome was attendance delays (in days), defined as the number of days between the date when an attendance took place vs the intended date as defined by the latest clinically appropriate date noted at the preceding attendance. In the base case, we allowed for a 4-week flexibility period to account for non-problematic delays; for example, patients could request to reschedule an appointment to accommodate holidays or changes in their schedules. Attendances that occurred within the 4-week flexibility window were considered non-delayed and were therefore modified to zero (sensitivity analyses assessed robustness of the results to both this assumption and to shortening the window, see online supplemental material). The reason for modifying to zero in the base case was that if the appointment was not considered delayed, it would be fairer to consider it zero rather than allowing appointments that took place for reasons of convenience in the days just before or just after the LCAD to affect the mean delay days, in case there was any systematic preference for shifting appointments a few days earlier or a few days later.

### Statistical analysis

We performed an interrupted time series analysis to estimate the impact of opening the HERCULES asynchronous review clinic on glaucoma and medical retina attendance delays.^15^ We fitted heteroskedasticity robust population-averaged linear panel data models, which estimate average effects rather than individual-specific effects while accounting for variability due to individual effects.^16^ We controlled for the spring 2020 COVID-19 national lockdowns and patient sociodemographic characteristics, which included the level of deprivation according to the Index of Multiple Deprivation (IMD).

The conceptual model was as follows:


(1) $$Y_{t}=b_{0}+b_{1}T_{1}+b_{2}T_{2}+b_{3}T_{3}+b_{i}X_{1}+e_{t}$$

where yt_t_ was average attendance delays, in days, over time_t_. time was specified as a linear spline with kinks marking the first covid-19 national lockdown in the week of 16 march 2020 (week 92 in our observation period) and the opening of the asynchronous review clinic at the intervention site in the week of 11 october 2021 (week 169). while the uk officially began the first lockdown on 23 march 2020, some changes in people’s behaviour preceded the official lockdown declaration. the greater london authority covid-19 mobility report highlighted a sharp decrease in mobility to parks and workplaces around 16 march 2020, in line with the prime minister’s recommendation for people to stop non-essential contact and travel on 16 march 2020.^17 18^ b1_1_ was the *attendance delay trend* before the first covid-19 lockdown (T_i_); b _2_ was the *trend* after first national covid-19 lockdown (T_3_); b _3_ was the *trend* after opening the virtual review hub at the intervention site (T_3_). b_i_ was a coefficient vector of sociodemographic characteristics X _i_ (potential residual confounders); b_i_ was expressed as the *average attendance delay difference (days*) between levels within each characteristic over the observed period while e_t_ represented the error term.

This report presents four key models (see online supplemental materials for further related models) conducted in Stata 18^19^ and reported at the 95% confidence level.

- Model 1 (base model): follows the conceptual model highlighted above in Equation 1, with non-problematic delays modified to zero.
- Model 2: tested for seasonality using a dummy for winter months (winter encompassing December, January and February of each year). We expected that appointment delays would increase in the winter months.
- Model 3: examined the impact of opening a smaller asynchronous review clinic (hereafter referred to as ‘precursor site’) at a separate site closer to the main hospital site on 10 February 2021 to provide similar outpatient services for monitoring patients with glaucoma and medical retina conditions.^20^
- Model 4: tested whether delay trends were similar among patients that attended the main facility vs all other sites. We first stratified the sample and fitted Model 2 for each subsample and the results justified the interaction of facility site and disease (see online supplemental table 7).

### Patient and public involvement

This analysis has benefited from the inclusion of a Patient and Public Involvement and Engagement (PPIE) member (co-author SNa) in our core research team who helped refine the research question and the interpretation of results for non-technical audiences, along with the rest of the research team. Other PPIE representation came from the Moorfields Biomedical Research Council (BRC) PPIE group leads, co-authors JC and HB, who also helped in interpreting the findings and ensuring they were presented in a more relevant and impactful manner for end users. The HERCULES project was reviewed and supported by Glaucoma UK, The Macular Society, Diabetes UK and Moorfields Eye Charity, all of which have a mission to promote interests of patients with eye disease.

## Results

**Figure 1:**
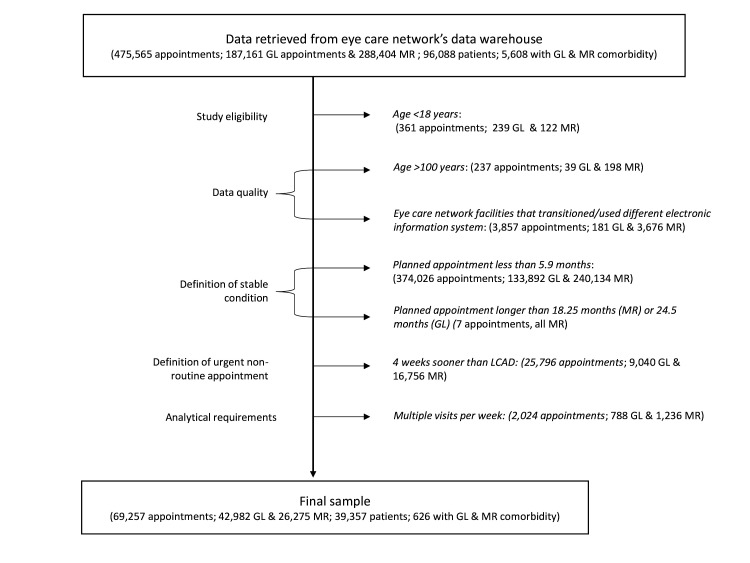
Flow chart of the data cleaning process. Patient records included demographic details such as year of birth, gender and ethnicity, alongside attendance-specific information such as attendance dates, intended follow-up schedules, diagnosed condition and healthcare facility details (table 1). GL, glaucoma; LCAD, latest clinically appropriate date; MR, medical retina.

Having followed the data cleaning process highlighted in figure 1, our final and analysed sample had 69 257 attendances, from 39 357 patients, across 13 sites within the eye care facility network (table 1). Glaucoma accounted for 62% (n=42 982) of attendances while 38% (n=26 275) were medical retina; a few patients (0.9%, n=626) attended for both glaucoma and medical retina (figure 1). There was an even split between males and females (51%, n=35 048) and 49%, n=34 204) and patients older than 65 years accounted for 54% (n=37 824) of attendances while those in the more deprived half of the population (IMD deciles 1 to 5) accounted for 53% (n=37 014) of attendances. Furthermore, the main facility site in central London accounted for 39% (n=27 339) of attendances, while the intervention site and all other sites accounted for 5% (n=3137) and 56% (n=38 781) respectively.

**Table 1:** Summary of attendances by patient characteristic

	Attendances (column %)		
	Glaucoma	Medical retina	All
N	42 982	26 275	69 257
Mean delay days over the observed period (SD)	90 (137)	87 (132)	89 (135)
Gender (%)			
Female	21 574 (50%)	12 630 (48%)	34 204 (49%)
Male	21 404 (50%)	13 644 (52%)	35 048 (51%)
Unknown	4 (0%)	1 (0%)	5 (0%)
Age group			
Mean age (SD)	66 (15)	63 (16)	65 (15)
Median age (Q1, Q3)	68 (56, 77)	64 (53, 74)	66 (55, 76)
18–24 years (n, %)	497 (1%)	338 (1%)	835 (1%)
25–34 years (n, %)	1071 (2%)	1021 (4%)	2092 (3%)
35–44 years (n, %)	2271 (5%)	2009 (8%)	4280 (6%)
45–54 years (n, %)	5345 (12%)	3990 (15%)	9335 (13%)
55–64 years (n, %)	8852 (21%)	6039 (23%)	14 891 (22%)
65–74 years (n, %)	11 164 (26%)	6312 (24%)	17 476 (25%)
75–84 years (n, %)	9605 (22%)	4796 (18%)	14 401 (20%)
85–100 years (n, %)	4177 (10%)	1770 (7%)	5947 (9%)
Indices of multiple deprivation (IMD) decile (n, %)			
1 (most deprived)	982 (2%)	639 (2%)	1621 (2%)
2	5140 (12%)	3209 (12%)	8349 (12%)
3	6387 (15%)	4120 (16%)	10 507 (15%)
4	5297 (12%)	3427 (13%)	8724 (13%)
5	4639 (11%)	3174 (12%)	7813 (11%)
6	5110 (12%)	3396 (13%)	8506 (12%)
7	4270 (10%)	2637 (10%)	6907 (10%)
8	3897 (9%)	1999 (8%)	5896 (9%)
9	3749 (9%)	2016 (8%)	5765 (9%)
10 (least deprived)	3303 (8%)	1492 (6%)	4795 (7%)
Missing	208 (0%)	166 (1%)	374 (1%)
Eye care facility (n, %)			
Main site	17 923 (42%)	9416 (36%)	27 339 (39%)
Intervention site	1620 (4%)	1517 (6%)	3137 (5%)
All other sites	23 439 (55%)	15 342 (58%)	38 781 (56%)
‘Ethnicity’ (n, %)			
African	2758 (6%)	849 (3%)	3607 (5%)
Any other Asian background	1308 (3%)	1296 (5%)	2604 (3%)
Any other Black background	420 (1%)	166 (1%)	586 (0%)
Any other White background	1794 (4%)	891 (3%)	2685 (3%)
Any other ethnic group	5796 (13%)	4361 (17%)	10 157 (14%)
Any other mixed background	160 (0%)	72 (0%)	232 (0%)
Bangladeshi	647 (2%)	579 (2%)	1226 (1%)
British	12 538 (29%)	4763 (18%)	17 301 (24%)
Caribbean	2385 (6%)	1114 (4%)	3499 (5%)
Chinese	265 (1%)	137 (1%)	402 (0%)
Indian	3850 (9%)	3354 (13%)	7204 (10%)
Irish	584 (1%)	250 (1%)	834 (1%)
Pakistani	698 (2%)	744 (3%)	1442 (2%)
White and Asian	65 (0%)	40 (0%)	105 (0%)
White and Black African	83 (0%)	50 (0%)	133 (0%)
White and Black Caribbean	142 (0%)	71 (0%)	213 (0%)
Not stated	7655 (18%)	6910 (26%)	14 565 (21%)
Unknown	1834 (4%)	628 (2%)	2462 (3%)

N, n, observations; Q1, lower quartile; Q3, upper quartile.

### Interrupted time series analysis results

**Figure 2:**
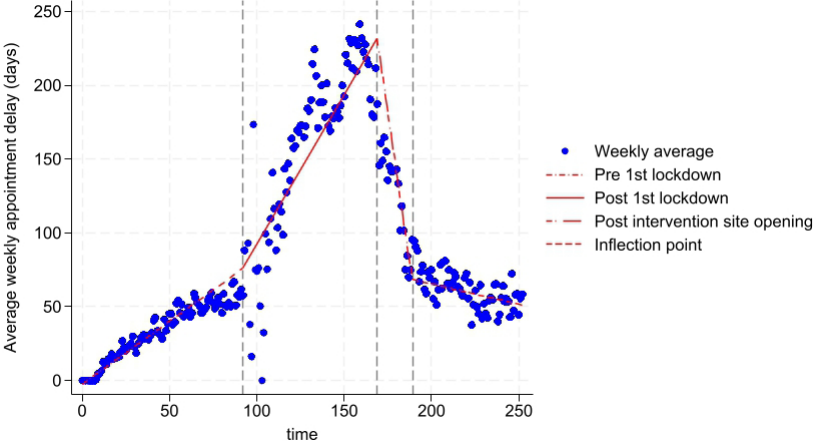
Overview of mean appointment delays (days) per week and trends estimated in Model 1 from the start of the observation period June 2018 (time=0 weeks) to April 2023 (time=252 weeks), including the first interruption (16 March 2020; time=92 weeks) which depicted the first COVID-19 national lockdown and the second interruption (11 October 2021; time=169 weeks) which depicted the opening of the virtual review hub at the intervention site shopping centre. The inflection point was 5 months post the intervention site opening.

Appointment attendance delays had been increasing prior to the intervention at 0.9 days per week (95% CI, 0.8 to 0.9) pre-COVID-19 lockdown and 2.0 days per week (95% CI, 2.0 to 2.0) after the lockdown (figures2  3, and Model 1 in table 2). However, opening the asynchronous review clinic at the intervention site corresponded with a sharp decrease in appointment delays, to 8.1 days per week (95% CI, 8.1 to 8.2) for the first 5 months, slowing to 0.3 days per week (95% CI, 0.3 to 0.3) thereafter.

**Figure 3:**
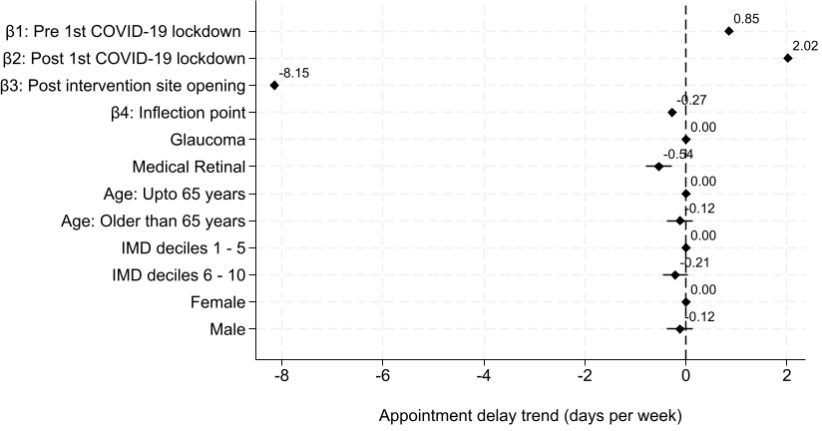
A forest plot showing appointment attendance delay trends estimated using Model 1, our base case model. Attendance delays were increasing pre-COVID-19 lockdown and the delays worsened post first COVID-19 lockdown. They decreased sharply after the intervention site opening, then this slowed, potentially after the backlog was cleared.

**Table 2:** Model results

	Model 1	Model 2	Model 3	Model 4
	β/(CI 95)	β/(CI 95)	β/(CI 95)	β/(CI 95)
β_1_: pre first COVID-19 lockdown	0.9***	0.8***	0.8***	0.8***
	(0.8, 0.9)	(0.8, 0.9)	(0.8, 0.8)	(0.8, 0.9)
β_2_: post first COVID-19 lockdown	2.0***	2.0***	2.2***	2.0***
	(2.0, 2.0)	(2.0, 2.0)	(2.2, 2.2)	(2.0, 2.0)
β_3_: post intervention site opening	−8.1***	−8.2***	−6.4***	−8.2***
	(−8.2, −8.1)	(−8.2, −8.1)	(−6.5, −6.4)	(−8.2, −8.1)
β_4_: inflection point	−0.3***	−0.3***	−0.7***	−0.3***
	(−0.3, −0.3)	(−0.3, −0.3)	(−0.7, −0.7)	(−0.3, −0.3)
Precursor site opening			(omitted)	
Disease:				
Glaucoma	Reference	Reference	Reference	
Medical retina	−0.5***	−0.5***	−0.2	
	(−0.8, −0.3)	(−0.8, −0.3)	(−0.5, 0.0)	
Age (at appointment):				
Up to 65 years old	Reference	Reference	Reference	
Older than 65 years	−0.1	−0.1	−0.1	
	(−0.4, 0.1)	(−0.4, 0.2)	(−0.4, 0.1)	
IMD decile:				
Ranges 1 to 5	Reference	Reference	Reference	
Ranges 6 to 10	−0.2	−0.2	−0.3*	
	(−0.5, 0.0)	(−0.5, 0.0)	(−0.5, 0.0)	
Gender				
Female	Reference	Reference	Reference	
Male	−0.1	−0.1	−0.1	
	(−0.4, 0.1)	(−0.4, 0.1)	(−0.3, 0.1)	
Winter:				
Non-winter months		Reference		Reference
Winter months		1.4***		1.4***
		(1.2, 1.7)		(1.2, 1.7)
Disease-site interaction				
Glaucoma (non-main facility sites)				Reference
Glaucoma (main facility site)				0.1
				(−0.2, 0.4)
Medical retina (non-main facility sites)				−1.3***
				(−1.6, −0.9)
Medical retina (main facility site)				0.9***
				(0.5, 1.3)
Constant	−2.1***	−2.2***	−0.6***	−2.5***
	(−2.4, −1.8)	(−2.6, −1.9)	(−0.9, −0.3)	(−2.8, −2.2)
N	68 878	68 878	68 878	69 257
BIC	585 311.4	585 231	575 106	588 327.5
Adj.R^2^	93.5%	93.5%	94.4%	93.5%
RSS	19 747 911.5	19 721 783	17 028 305.5	19 798 169.3
RMSE	16.9	16.9	15.7	16.9

95% CIs in brackets.

*p<0.1, ***p<0.01.

Adj.R2, adjusted R squared; BIC, Bayesian Information Criterion; IMD, Index of Multiple Deprivation; RMSE, root mean squared error; RSS, residual sum of squares; β, estimated coefficients.

When considering the coefficients of the covariates in the models, Model 1 further highlighted that medical retina attendances were delayed, on average, by 0.5 (95% CI, 0.3 to 0.8) days less than glaucoma appointment delays, over the observed period. The difference in average attendance delays was not statistically different by age group (≤65 years vs >65 years), IMD decile (grouped as 1 to 5 (more deprived half) vs 6 to 10 (less deprived half)) or gender.

The base case model (Model 1) explained 93.5% of the variation in appointment delays. However, it used only 68 878 observations because 374 observations missed an IMD decile, and 5 observations had unknown gender (table 1), therefore excluded from the analysis. This represented only 0.5% of the data so was considered not to be influential on the overall model.

The impact of opening the asynchronous review clinic was not sensitive to the inclusion of a winter dummy term, but we found that winter attendances were on average delayed by 1.4 (95% CI, 1.2 to 1.7) days more compared with non-winter attendances over the observed period (Model 2 table 2).

However, the impact was sensitive to the inclusion of an additional kink reflecting the opening of a similar asynchronous review clinic, the precursor clinic, at a site closer to the main facility site a few months before the intervention site opened. As result of this additional kink, the impact of our intervention decreased from ~8 days per week to 6.4 days per week (95% CI, 6.4 to 6.5) (table 2). However, the kink term was itself omitted in Model 3 due to collinearity, meaning it could be expressed as a perfect linear combination of at least one of the included explanatory variables.^21^

Lastly, when considering an interaction term between disease and whether attendances took place at the main facility site or elsewhere, and removing the age, gender and deprivation covariates, we found insignificant differences in average delays between glaucoma attendances at the main facility site and at all other sites (reference category=glaucoma at all other sites); however, medical retina attendances at all other sites experienced 1.3 (95% CI, 1.6 to 1.9) days less delays relative to glaucoma attendances at non-main sites. Finally, medical retina attendances at the main site experienced 0.9 (95% CI 0.5 to 1.3) days more delays over the observed period, relative to the reference, as seen in Model 4 (table 2). Note that Model 4 had 69 257 observations, as it was exploring the relationship between disease and whether the attendance took place at the main facility or elsewhere, and did not include demographic covariates which had missing items as described above.

## Discussion

### Statement of principal findings

Attendance delays for glaucoma and medical retina were already increasing by 0.9 days per week before the COVID-19 pandemic, worsening to 2.0 days per week after the first COVID-19 lockdown. This sharp increase in attendance delays corresponded with the promulgation of national COVID-19 guidelines that called for service structuring and limited mobility.^22^ For instance, the eye hospital network initially restricted its services to cater for emergency and sight-threatening conditions and triaged cases from its 26 sites across Greater London to the main facility during the COVID-19 lockdown, which increased delays for non-emergency cases.^23^ This was also evident at the national level. For example, data from Specsavers’ Hindsight Report (2021), which conducts 46% of eye checks in the UK, estimated that 4.3 million eye tests were not delivered in 2020 in the UK compared to 2019, representing a 23% decline.^24^ Using NHS Digital’s Hospital Episode Statistics, the report further highlighted a decrease in ophthalmology treatments: outpatient attendances and day case procedures declined by 36% and 45% respectively between March and December 2020 compared with the same period in 2019.^25^

The asynchronous review clinic at the intervention site changed the eye hospital network’s attendance delay trajectory; after opening, delays decreased by 8.1 days per week. We believe the inflection in rate of decrease 5 months after opening reflects the time it took for the hospital network to clear the COVID-19-induced backlog. The network had initiated various strategies to address attendance delays, including opening a precursor asynchronous review clinic in February 2021. This clinic was designed in line with our group’s previous research and based on operating concepts of the first layout iteration at the intervention site.^12^ However, the precursor clinic did not have a strong impact on the trajectory of attendance delays, probably because it operated below its designed capacity. Other network-wide interventions included the introduction of rapid glaucoma assessment clinics in October 2020 to re-stratify previously ‘low risk’ patients who would otherwise be subject to substantial further appointment delays. This initiative might partially explain the different trend direction depicted in Model 4 between medical retinal and glaucoma attendances at the main hospital site and other sites (table 2). Various interventions have been implemented at the national level to address appointment backlogs; these include community diagnostic centres, pre-referral advanced diagnostic imaging and engaging independent sector providers.^26 27^ However, the impact of these strategies on appointment attendance delays is not well documented. To the best of our knowledge, this is the first study that examines the impact of a new model on appointment attendance delays.

We did not find differences in attendance delays by age, gender or level of deprivation (IMD) across the eye hospital network. However, patients with accessibility needs such as wheelchair users were not triaged to the intervention clinic, although some wheelchair users did attend. This raises questions from equity perspective. The Association of Optometrists has proposed that community-based service delivery models should address accessibility barriers observed in hospital sites, including for the elderly and accompanied patients.^28^ The capacity needs and economic case for replication of hospital-level facilities within asynchronous review clinics should be examined in future research. While the delay differences are statistically different by diagnosed disease and winter vs not winter, they are practically insignificant as they were less than 2 days.

### Strengths and limitations

First, our analysis lacks external controls as we had data from one eye hospital network; therefore, cannot compare our results against national or regional trends. However, the methods we have used are robust for evaluating impact when randomisation is not possible.^29^ Second, while the dataset included all appointment attendees and therefore represented a wide range of patient demographic groups, incomplete data and ambiguous category wording on ethnicity impeded further subgroup analyses and closer comparison against expected population values. However, since ethnicity often correlates with IMD,^30^ which we found not to be an important explanatory variable, we consider that the impact of the incomplete data was limited. Lastly, we have focused on glaucoma and medical retina patients but in 2022 the hospital network expanded the scope of activities at the intervention site to include cataract patients and newly referred patients; therefore, this analysis might underestimate the overall contribution to reducing ophthalmic appointment delays across the network. A further limitation is that this observational retrospective analysis focuses on attendance delays and does not explicitly consider patient outcomes such as eye health or broader health or quality of life nor does it consider direct quality measures such as the agreement between scans performed by technicians or performed by traditional staff types at the main hospital site. These aspects are all outside the scope of this work, but they and other parts of the patient pathway would be important factors to consider in related and future work.

### Policy implications and opportunities for future research

Our findings highlight this community-based service delivery model’s promise in addressing the significant healthcare service delivery bottlenecks in ophthalmology, amid NHS workforce and estate pressures. This service delivery model could potentially be scaled to other high-volume low-complexity services in the NHS and is especially timely, given the current focus on expanding community-based care, which is a priority of the new government and a recommendation from the recently published Lord Darzi report.^1^ Nonetheless, scaling up this approach and replicating its successes require an understanding of the operational and system-level arrangements that contributed to success. For instance, the precursor clinic did not yield similar reductions in attendance delays. Qualitative research is forthcoming from our group regarding these aspects. Furthermore, our work does not provide evidence on the cost-effectiveness of asynchronous review clinics. This is important for future research as it could demonstrate value for money for the NHS.

In summary, our intervention corresponded with a significant decrease in appointment delays for patients with stable chronic eye diseases. We believe, therefore, that this model could be used more widely across the country in high-volume low-complexity contexts where services could be amenable to partial delivery using a newly recruited and trained healthcare-naïve technician cadre.

### Dissemination to participants and related patient and public communities

We will disseminate our findings to decision makers, service staff and the wider community at the large London teaching hospital trust and other relevant healthcare planners in the UK to inform the planning of high-volume low-complexity services. We will also share our findings with the public through our existing networks via the Moorfields Biomedical Research Centre (BRC) Patient and Public Involvement and Engagement (PPIE) group and eye charity lists, as well as press releases and social media engagement, and with the academic community at scientific conferences.

### Participant consent

This study uses retrospective, routinely collected electronic attendance records that were anonymised prior to analysis. In accordance with the North East - York Research Ethics Committee ethics approval for the HERCULES study (Reference 21/NE/0164), no identifiable personal information was accessed by analysis team for this work and informed consent was not necessary.

### Contributions and data access

DJF provided the initial dataset via secure file transfer into the UCL Data Safe Haven (ISO/IEC 27001:2013), a secure platform for storing, handling and analysing identifiable data, then SNd, JCBA and CSC analysed the anonymised dataset within the Data Safe Haven.

## Supplementary material

10.1136/bmjopen-2025-098820 online supplemental file 1

## Data Availability

No data are available.
