# Supplementary material for: Impact of a community-based asynchronous review clinic on appointment attendance delays across an eye hospital network in London, UK: an interrupted time series analysis
Source: BMJ Open. 2025 Jul 15;15(7):e098820. doi: 10.1136/bmjopen-2025-098820 (PMC12273167; doi:10.1136/bmjopen-2025-098820)
Supplement: online supplemental file 1 [file bmjopen-15-7-s001.docx]

Supplemental Material for:

Impact of a Community-Based Asynchronous-Review Clinic on Appointment Attendance Delays Across an Eye Hospital Network in London, UK

Table of Contents

[Sensitivity to different thresholds for defining problematic attendance delays. 2](#_Toc174020253)

[Sensitivity of base model to exclusion of weeks with fewer observations 4](#_Toc174020254)

[Impact of virtual-review clinic on attendance delays by sociodemographic characteristics 6](#_Toc174020255)

[Sub-sample comparison between main hospital site and non-main hospital site attendances 9](#_Toc174020256)

[Comparing methodological approaches (OLS vs GEE) 11](#_Toc174020257)

[Members of the HERCULES Research Consortium 14](#_Toc174020258)

**Introduction**

This supplemental document provides detailed analyses to support the findings presented in the main manuscript. First, we assessed how varying the thresholds for what constitutes a problematic attendance delay impacts the results. Different cut-off points are explored to ensure robustness of the main findings. Secondly, we evaluated the sensitivity of the base model to the exclusion of weeks that had fewer observations understand the stability and reliability of the results when data sparsity is an issue. Thirdly, we examined the impact of the virtual-review clinic on attendance delays through the lens of various sociodemographic characteristics to identify any disparities or differential effects across different population subgroups. Fourth, we assessed the impact of interventions across the hospital network sites, comparing the main hospital site against all other sites. Lastly, we compared two widely used methodological approaches—ordinary least squares (OLS) and Generalised Estimating Equations (GEE)—to highlight any differences in results and the appropriateness of each method given the structure of our data.

# **Sensitivity to different thresholds for defining problematic attendance delays.**

Not all attendance delays are problematic and reflect inefficiencies in the healthcare system, instead they can reflect flexibility required by patients, e.g. changes in schedules due to vacations or other minor changes in personal circumstances. In the base model, we allowed for a four-week flexibility period to account for non-problematic delays. This effectively meant appointments seen sooner than four weeks early were regarded as indicative of non-stable disease and therefore removed from the sample. Appointments seen within the four-week flexibility window were considered non-delayed and were effectively modified to zero. Supplemental models (SM) 1 and 2 examine the base model’s sensitivity to the applied inconsequential-delays threshold using cut-off points at two (SM 1) and three weeks (SM2) whilst SM3 examined sensitivity to modifying non-problematic delays to zero.

The base model was not sensitive to the two-week and three-week thresholds for problematic delays we tested. SM1 and SM2 both highlighted that attendance delays decreased by 8.2 days per week (d/w) after the opening of the virtual-review clinic at the intervention site. Furthermore, the base model was not sensitive to modifying attendance delays to zero for appointments that were early or delayed by at most four weeks. SM3 highlighted that opening the virtual-review-clinic corresponded with 8.0 d/w reduction in attendance. The estimated coefficients’ 95% confidence intervals in SM1, SM2, and SM3 intersect with the base model’s confidence intervals, thus providing no evidence of statistically significant difference. Additionally, these models had comparable fit to the data as highlighted by the small differences in BIC, Adj.R^2^ , RRSS, and RMSE.

Supplemental Table 1. Sensitivity of base case model to different cut-offs for defining problematic appointment delays

|  | Base Model | SM1  (2 weeks) | SM2  (3 weeks) | SM3  (non-zero) |
| --- | --- | --- | --- | --- |
| β_1_: Pre 1st COVID-19 lockdown | 0.9*** | 0.8*** | 0.9*** | 0.8*** |
|  | [0.8, 0.9] | [0.8, 0.9] | [0.8, 0.9] | [0.8, 0.8] |
| β 2: Post 1st COVID-19 lockdown | 2.0*** | 2.1*** | 2.0*** | 2.0*** |
|  | [2.0, 2.0] | [2.0, 2.1] | [2.0, 2.0] | [2.0, 2.0] |
| β3: Post intervention site opening | -8.1*** | -8.2*** | -8.2*** | -8.0*** |
|  | [-8.2, -8.1] | [-8.2, -8.1] | [-8.2, -8.1] | [-8.1, -8.0] |
| β 4: Inflection point | -0.3*** | -0.3*** | -0.3*** | -0.3*** |
|  | [-0.3, -0.3] | [-0.3, -0.3] | [-0.3, -0.3] | [-0.3, -0.3] |
| Disease: |  |  |  |  |
| Glaucoma | Reference | Reference | Reference | Reference |
| Medical Retina | -0.5*** | -0.6*** | -0.6*** | -0.5*** |
|  | [-0.8, -0.3] | [-0.8, -0.3] | [-0.9, -0.3] | [-0.8, -0.3] |
| Age (at appointment): |  |  |  |  |
| Up to 65 years old | Reference | Reference | Reference | Reference |
| Older than 65 years | -0.1 | -0.1 | -0.1 | -0.1 |
|  | [-0.4, 0.1] | [-0.3, 0.2] | [-0.3, 0.2] | [-0.4, 0.1] |
| IMD decile: |  |  |  |  |
| Ranges 1 to 5 | Reference | Reference | Reference | Reference |
| Ranges 6 to 10 | -0.2 | -0.2 | -0.2 | -0.2 |
|  | [-0.5, 0.0] | [-0.5, 0.0] | [-0.5, 0.0] | [-0.5, 0.0] |
| Gender |  |  |  |  |
| Female | Reference | Reference | Reference | Reference |
| Male | -0.1 | -0.1 | -0.1 | -0.1 |
|  | [-0.4, 0.1] | [-0.4, 0.1] | [-0.4, 0.1] | [-0.4, 0.1] |
| Constant | -2.1*** | 1.7*** | -0.5** | 2.4*** |
|  | [-2.4, -1.8] | [1.4, 2.1] | [-0.8, -0.2] | [2.0, 2.7] |
| N | 68878 | 66605 | 67804 | 68878 |
| BIC | 585311.4 | 563551.2 | 575431.5 | 581537.4 |
| Adj.R^2^ | 93.5% | 93.9 % | 93.6 % | 93.6% |
| RSS | 19747911.5 | 18407182.3 | 19224802 | 18694992.4 |
| RMSE | 16.9 | 16.6 | 16.8 | 16.5 |

β = estimated coefficients; SM = supplemental model; BIC = Bayesian Information Criteria; Adj.R^2^ = adjusted residual square; RSS = residual sum squares; RMSE = Root Mean Squared Error; 95% confidence intervals in brackets

* p<0.1, ** p<0.05, ***p<0.01

# **Sensitivity of base model to exclusion of weeks with fewer observations**

Our dataset had an uneven distribution of appointments over the observed period, June 2018 to April 2023. As Supplemental Figure 1 shows, recorded appointments were sparce towards the beginning of our observation period, June to August 2018 and, as expected, immediately after the first COVID-19 national lockdown. Sparsity at the beginning of our observation coincides with the implementation of new data capturing systems and changes in clinical referral letter formats, which impacted data collection.


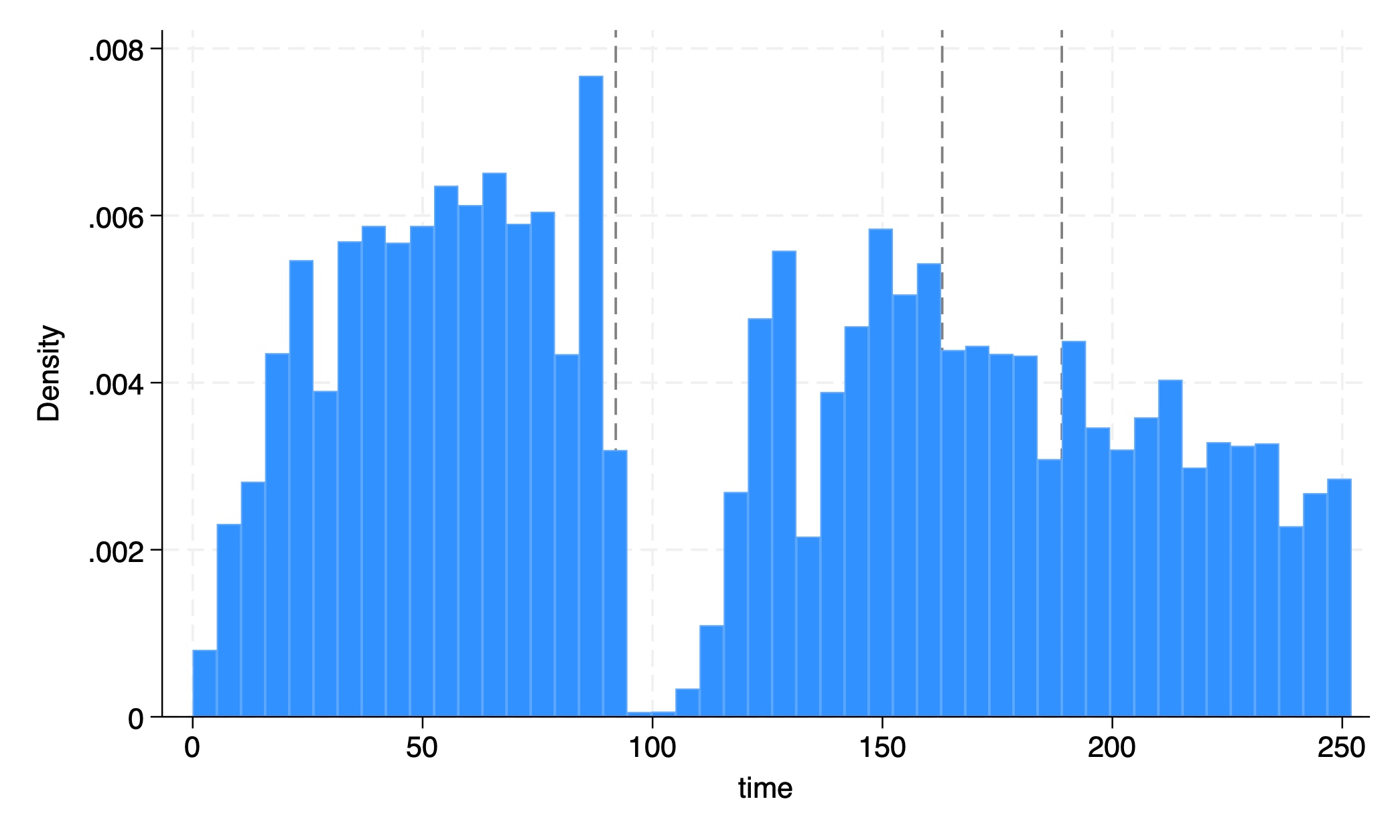


Supplemental Figure 1. The distribution of appointments was not even across the observed period.

We assessed the base model’s sensitivity to missingness by dropping observations in weeks with fewer than 50 appointments per week. We assumed that the missing observations were missing completely at random because lockdown and changes in information systems would have affected patients equally

Supplemental Table 2. Sensitivity of base case model to exclusion of weeks with sparse observations

|  | **Base** | **SM4**  **(exclude weeks with <50 obs.)** |
| --- | --- | --- |
| β1: Pre 1st COVID-19 lockdown | 0.9*** | 0.9*** |
|  | [0.8, 0.9] | [0.8, 0.9] |
| β2: Post 1st COVID-19 lockdown | 2.0*** | 2.0*** |
|  | [2.0, 2.0] | [2.0, 2.0] |
| β3: Post intervention site opening | -8.1*** | -8.1*** |
|  | [-8.2, -8.1] | [-8.2, -8.1] |
| β4: Inflection point | -0.3*** | -0.3*** |
|  | [-0.3, -0.3] | [-0.3, -0.3] |
| Disease: |  |  |
| Glaucoma | Reference | Reference |
| Medical Retina | -0.5*** | -0.5*** |
|  | [-0.8, -0.3] | [-0.8, -0.3] |
| Age (at appointment): |  |  |
| Up to 65 years old | Reference | Reference |
| Older than 65 years | -0.1 | -0.1 |
|  | [-0.4, 0.1] | [-0.4, 0.1] |
| IMD decile: |  |  |
| Ranges 1 to 5 | Reference | Reference |
| Range 6 to 10 | -0.2 | -0.2 |
|  | [-0.5, 0.0] | [-0.5, 0.0] |
| Gender: |  |  |
| Female | Reference | Reference |
| Male | -0.1 | -0.1 |
|  | [-0.4, 0.1] | [-0.4, 0.1] |
| Constant | -2.1*** | -2.2*** |
|  | [-2.4, -1.8] | [-2.5, -1.9] |
| N | 68878 | 68651 |
| BIC | 585311.4 | 582870.3 |
| Adj.R^2^ | 93.5% | 93.5% |
| RSS | 19747912 | 19536456 |
| RMSE | 16.9 | 16.9 |

β = estimated coefficients; SM = supplemental model; BIC = Bayesian Information Criteria; Adj.R^2^ = adjusted residual square; RSS = residual sum squares; RMSE = Root Mean Squared Error; 95% confidence intervals in brackets

* p<0.1, ** p<0.05, ***p<0.01

# **Impact of virtual-review clinic on attendance delays by sociodemographic characteristics**

We also assessed whether the intervention’s impact on attendance delays was differentiated by condition, age group, level of deprivation, and gender. Summary tables 3,Supplemental Table *4*, 5, and Supplemental Table *6* suggested no statistically significant difference in effect; attendance delays decreased by 8.1 to 8.2 d/w (95% CI) across the assessed sociodemographic characteristics.

Supplemental Table 3. Impact of virtual-review clinic by disease

|  | Base | GL | MR |
| --- | --- | --- | --- |
| β_1_: Pre 1st COVID-19 lockdown | 0.9*** | 0.9*** | 0.8*** |
|  | [0.8,0.9] | [0.9,0.9] | [0.8,0.8] |
| β _2_: Post 1st COVID-19 lockdown | 2.0*** | 2.0*** | 2.0*** |
|  | [2.0,2.0] | [2.0,2.0] | [2.0,2.1] |
| β _3_: Post intervention site opening | -8.1*** | -8.1*** | -8.2*** |
|  | [-8.2, -8.1] | [-8.2, -8.1] | [-8.2, -8.1] |
| β _4_: Inflection point | -0.3*** | -0.3*** | -0.3*** |
|  | [-0.3, -0.3] | [-0.3, -0.3] | [-0.3, -0.3] |
| Constant | -2.5*** | -3.0*** | -1.7*** |
|  | [-2.7, -2.3] | [-3.3, -2.8] | [-2.0, -1.3] |
| N | 69257 | 42982 | 26275 |
| BIC | 588487.7 | 365409.4 | 223074.5 |
| Adj.R2 | 0.9 | 0.9 | 0.9 |
| RSS | 19856796.3 | 12371046 | 7471066.7 |
| RMSE | 16.9 | 17 | 16.9 |

β = estimated coefficients; BIC = Bayesian Information Criteria; Adj.R^2^ = adjusted residual square; RSS = residual sum squares; RMSE = Root Mean Squared Error; 95% confidence intervals in brackets

* p<0.1, ** p<0.05, ***p<0.01

Supplemental Table 4. Impact of virtual-review clinic by age group

|  | Base | ≤65 years old | >65 years old |
| --- | --- | --- | --- |
| β_1_: Pre 1st COVID-19 lockdown | 0.9*** | 0.9*** | 0.9*** |
|  | [0.8,0.9] | [0.8,0.9] | [0.8,0.9] |
| β _2_: Post 1st COVID-19 lockdown | 2.0*** | 2.0*** | 2.0*** |
|  | [2.0,2.0] | [2.0,2.0] | [2.0,2.0] |
| β _3_: Post intervention site opening | -8.1*** | -8.2*** | -8.1*** |
|  | [-8.2, -8.1] | [-8.2, -8.1] | [-8.2, -8.1] |
| β _4_: Inflection point | -0.3*** | -0.3*** | -0.3*** |
|  | [-0.3, -0.3] | [-0.3, -0.3] | [-0.3, -0.3] |
| Constant | -2.5*** | -2.7*** | -2.4*** |
|  | [-2.7, -2.3] | [-3.0, -2.3] | [-2.7, -2.1] |
| N | 69257 | 33073 | 36184 |
| BIC | 588487.7 | 282001.6 | 306495.2 |
| Adj.R^2^ | 0.9 | 0.9 | 0.9 |
| RSS | 19856796.3 | 9758625.7 | 10094555.4 |
| RMSE | 16.9 | 17.2 | 16.7 |

β = estimated coefficients; BIC = Bayesian Information Criteria; Adj.R^2^ = adjusted residual square; RSS = residual sum squares; RMSE = Root Mean Squared Error; 95% confidence intervals in brackets

* p<0.1, ** p<0.05, ***p<0.01

Supplemental Table 5. Impact of virtual-review clinic by level of deprivation

|  | Base | IMD  (ranges 1 to 5) | IMD  (ranges 6 to 10) |
| --- | --- | --- | --- |
| β_1_: Pre 1st COVID-19 lockdown | 0.9*** | 0.9*** | 0.8*** |
|  | [0.8, 0.9] | [0.8, 0.9] | [0.8, 0.9] |
| β _2_: Post 1st COVID-19 lockdown | 2.0*** | 2.0*** | 2.0*** |
|  | [2.0, 2.0] | [2.0, 2.0] | [2.0, 2.0] |
| β _3_: Post intervention site opening | -8.1*** | -8.2*** | -8.1*** |
|  | [-8.2, -8.1] | [-8.2, -8.1] | [-8.2, -8.1] |
| β _4_: Inflection point | -0.3*** | -0.3*** | -0.3*** |
|  | [-0.3, -0.3] | [-0.3, -0.3] | [-0.3, -0.3] |
| Constant | -2.5*** | -2.5*** | -2.6*** |
|  | [-2.7, -2.3] | [-2.8, -2.2] | [-2.9, -2.2] |
| N | 69257 | 37014 | 31869 |
| BIC | 588487.7 | 314925.8 | 270443.7 |
| Adj.R^2^ | 0.9 | 0.9 | 0.9 |
| RSS | 19856796.3 | 10724510 | 9029350.2 |
| RMSE | 16.9 | 17 | 16.8 |

β = estimated coefficients; BIC = Bayesian Information Criteria; Adj.R^2^ = adjusted residual square; RSS = residual sum squares; RMSE = Root Mean Squared Error; 95% confidence intervals in brackets

* p<0.1, ** p<0.05, ***p<0.01

Supplemental Table 6. Impact of virtual-review clinic by gender

|  | Base | Females | Males |
| --- | --- | --- | --- |
| β_1_: Pre 1st COVID-19 lockdown | 0.9*** | 0.9*** | 0.8*** |
|  | [0.8,0.9] | [0.8,0.9] | [0.8,0.9] |
| β _2_: Post 1st COVID-19 lockdown | 2.0*** | 2.0*** | 2.0*** |
|  | [2.0,2.0] | [2.0,2.0] | [2.0,2.0] |
| β _3_: Post intervention site opening | -8.1*** | -8.1*** | -8.1*** |
|  | [-8.2, -8.1] | [8.2, -8.1] | [-8.2, -8.1] |
| β _4_: Inflection point | -0.3*** | -0.3*** | -0.3*** |
|  | [-0.3, -0.3] | [0.3, -0.3] | [-0.3, -0.3] |
| Constant | -2.5*** | -2.6*** | -2.4*** |
|  | [-2.7, -2.3] | [3.0, -2.3] | [-2.7, -2.1] |
| N | 69257 | 34204 | 35048 |
| BIC | 588487.7 | 290297.8 | 298187.2 |
| Adj.R^2^ | 0.9 | 0.9 | 0.9 |
| RSS | 19856796.3 | 9702957.7 | 10150864.3 |
| RMSE | 16.9 | 16.8 | 17 |

β = estimated coefficients; BIC = Bayesian Information Criteria; Adj.R^2^ = adjusted residual square; RSS = residual sum squares; RMSE = Root Mean Squared Error; 95% confidence intervals in brackets

* p<0.1, ** p<0.05, ***p<0.01

# **Sub-sample comparison between main hospital site and non-main hospital site attendances**

In testing whether delay trends were similar amongst patients that attended the main hospital trust site versus all other trust sites (model 4), we first stratified the sample and fit model 2 for each subsample. The results below showed that over the observed period, average attendance delays for medical retina at non-main hospital sites were less by 1.3 days relative to glaucoma attendances whilst medical retinal attendances at the main hospital site delayed by 0.8 days more relative to glaucoma attendances. We used this finding as the basis for adding an interaction term between diagnosed condition and trust site in model 4. The table below also highlights that opening the virtual-review had a marginally higher impact at the main hospital site than at non-main hospital site. This result ought to be interpreted with caution as there are large sample size differences between the two (n=41763 vs n=27115)

Supplemental Table 7. Sub-sample comparison between main hospital site and non-main hospital sites

|  | Non-main hospital site | | Main hospital site | |
| --- | --- | --- | --- | --- |
| β_1_: Pre 1st COVID-19 lockdown | 0.9*** | | 0.8*** | |
|  | [0.9,0.9] | | [0.8,0.8] | |
| β _2_: Post 1st COVID-19 lockdown | 2.0*** | | 2.1*** | |
|  | [2.0,2.0] | | [2.1,2.1] | |
| β _3_: Post intervention site opening | -8.0*** | | -8.5*** | |
|  | [-8.0, -8.0] | | [-8.5, -8.4] | |
| β _4_: Inflection point | -0.3*** | | -0.3*** | |
|  | [-0.3, -0.3] | | [-0.3, -0.2] | |
| Disease: |  | |  | |
| Glaucoma |  | |  | |
| Medical Retina | -1.3*** | | 0.8*** | |
|  | [-1.7, -1.0] | | [0.4,1.2] | |
| Age: |  | |  | |
| Up to 65 years |  | |  | |
| Older than 65 | -0.1 | | 0.0 | |
|  | [-0.4, 0.3] | | [-0.3, 0.4] | |
| IMD decile: |  | |  | |
| Ranges 1 to 5 |  | |  | |
| Ranges 6 to 10 | -0.1 | | -0.3 | |
|  | [-0.4, 0.2] | | [-0.7, 0.1] | |
| Gender |  | |  | |
| Female | 0.0 | | -0.3 | |
| Male | [-0.3, 0.3] | | [-0.6, 0.1] | |
|  |  | |  | |
| Non winter months | | |  |  |
| Winter months | | | 1.3*** | 1.4*** |
|  | [1.0, 1.7] | | [1.0, 1.8] | |
| Constant | -3.0*** | | -1.2*** | |
|  | [-3.4, -2.5] | | [-1.7, -0.7] | |
|  |  | |  | |
| N | 41763 | | 27115 | |
| BIC | 357710.5 | | 226667.6 | |
| Adjusted R^2 | 0.9 | | 0.9 | |
| RSS | 12795322.4 | | 6754319.6 | |
| RMSE | 17.5 | | 15.8 | |
| β = estimated coefficients; BIC = Bayesian Information Criteria; Adj.R^2^ = adjusted R squared; RSS = residual sum squares; RMSE = Root Mean Squared Error; 95% confidence intervals in brackets  * p<0.1, ** p<0.05, ***p<0.01 | | | | |

# **Comparing methodological approaches (OLS vs GEE)**

Alternative to Ordinary Least Squares (OLS), interrupted time series can also be estimated using generalised estimation equations (GEE), which estimate population-averaged effects whilst accounting for within-group correlation. This is highly advantageous with correlated data with repeated measures. The OLS model on the other hand is simpler and suitable for independent data. Our data was less likely to be correlated at the patient level because of the sporadic attendances common amongst patients with stable glaucoma and medical retina disease. We still tested whether OLS and GEE approaches yielded different intervention impact estimates. We found that the OLS and GEE models yielded similar estimates (~8 d/w). Contrary to the OLS model, the GEE model suggested that the average difference in attendance delays between males and females was significantly different (at 99% confidence level). The GEE model also yielded results with wider 95% confidence intervals (Supplemental Table 8), larger mean, and standard deviation (Supplemental Table 9).

Supplemental Table 8. Comparison of OLS and GEE models

|  | **Base (OLS)** | **GEE** |
| --- | --- | --- |
| β_1_: Pre 1st COVID-19 lockdown | 0.9*** | 0.9*** |
|  | [0.8, 0.9] | [0.8, 0.9] |
| β _2_: Post 1st COVID-19 lockdown | 2.0*** | 2.0*** |
|  | [2.0, 2.0] | [2.0, 2.1] |
| β _3_: Post intervention site opening | -8.1*** | -8.2*** |
|  | [-8.2, -8.1] | [-8.5, -7.9] |
| β _4_: Inflection point | -0.3*** | -0.3*** |
|  | [-0.3, -0.3] | [-0.4, -0.2] |
| Disease: |  |  |
| Glaucoma | Reference | Reference |
| Medical Retina | -0.5*** | -2.9** |
|  | [-0.8, -0.3] | [-4.8, -1.0] |
| Age: |  |  |
| Up to 65 years | Reference | Reference |
| Older than 65 | -0.1 | 1.8 |
|  | [-0.4, 0.1] | [-0.0, 3.7] |
| IMD decile: |  |  |
| Ranges 1 to 5 | Reference | Reference |
| Ranges 6 to 10 | -0.2 | -0.6 |
|  | [-0.5, 0.0] | [-2.4, 1.3] |
| Gender |  |  |
| Female | Reference | Reference |
| Male | -0.1 | -4.0*** |
|  | [-0.4, 0.1] | [-5.8, -2.1] |
| Constant | -2.1*** | -0.9 |
|  | [-2.4, -1.8] | [-3.1, 1.4] |
| N | 68878 | 68878 |
| BIC | 585311.4 |  |
| Adj.R2 | 93.5% |  |
| RSS | 19747911.5 |  |

β = estimated coefficients; BIC = Bayesian Information Criteria; Adj.R^2^ = adjusted residual square; RSS = residual sum squares; RMSE = Root Mean Squared Error; 95% confidence intervals in brackets

* p<0.1, ** p<0.05, ***p<0.01


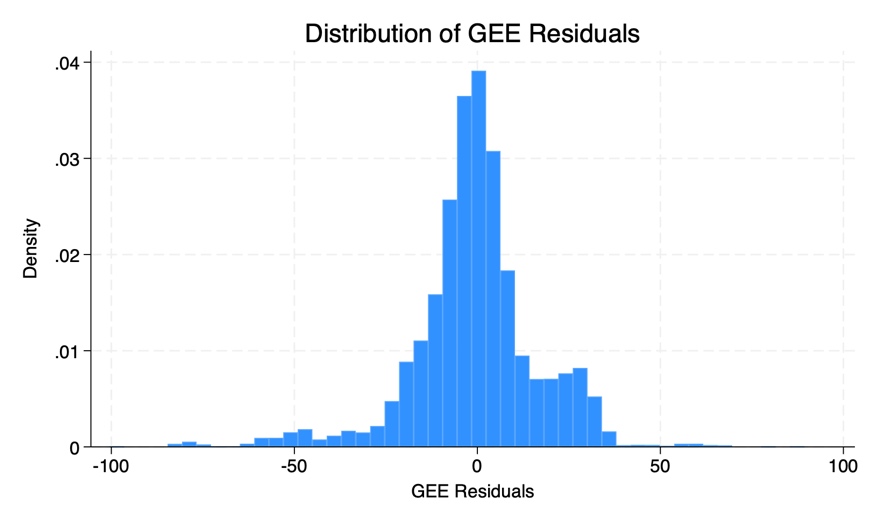


Supplemental Figure 2. Distribution of GEE residuals


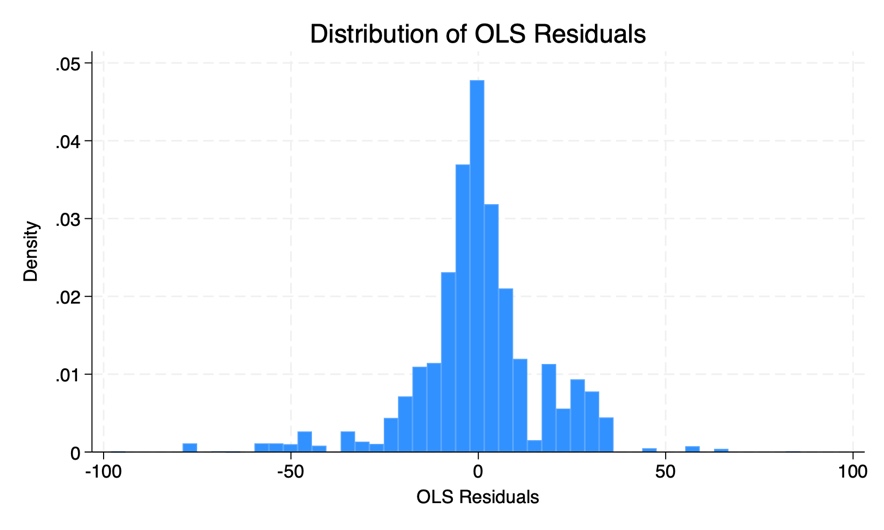


Supplemental Figure 3. Distribution of OLS residuals

Supplemental Table 9. Summary of OLS and GEE residuals

| Variable | Observations | Mean | Standard deviation | Minimum | Maximum |
| --- | --- | --- | --- | --- | --- |
| GEE errors | 68,878 | -.7 | 17.1 | -100.5 | 89.4 |
| OLS errors | 68,878 | 1.48e-15 | 16.9 | -98.0 | 85.9 |

# **Members of the HERCULES Research Consortium**

**Supplemental Table 10.** Members of the HERCULES Research Consortium

| First name | Last name | Affiliation |
| --- | --- | --- |
| Eleanor | Dean | Akeso |
| Olivia | Jeffrey | Akeso |
| Chris | Robson | Akeso |
| Nadine | Abdelgalil | Moorfields NHS FT |
| Joy | Adesanya | Moorfields NHS FT |
| Samiul | Alom | Moorfields NHS FT |
| Stacey | Angus | Moorfields NHS FT |
| Muna | Ayah | Moorfields NHS FT |
| Helen | Baker | Moorfields NHS FT |
| Connor | Beddow | Moorfields NHS FT |
| Paul | Cartwright | Moorfields NHS FT |
| Elisha | Chung | Moorfields NHS FT |
| George | Damianidis | Moorfields NHS FT |
| Sarah | Davies | Moorfields NHS FT |
| Sandi | Drewett | Moorfields NHS FT |
| Matala | Dyke | Moorfields NHS FT |
| Sherene | Ettiene | Moorfields NHS FT |
| Clare | Feasby | Moorfields NHS FT |
| Declan | Flanagan | Moorfields NHS FT |
| Dun Jack | Fu | Moorfields NHS FT |
| Gus | Gazzard | Moorfields NHS FT |
| Simranjit | Gill | Moorfields NHS FT |
| Steven | Gill | Moorfields NHS FT |
| Robin | Hamilton | Moorfields NHS FT |
| Nick | Hardie | Moorfields NHS FT |
| Jamie | Henderson | Moorfields NHS FT |
| Lesley | Henry | Moorfields NHS FT |
| Hari | Jayaram | Moorfields NHS FT |
| Aadil | Kazi | Moorfields NHS FT |
| Peng Tee | Khaw | Moorfields NHS FT |
| Chris | Leak | Moorfields NHS FT |
| Richard | Lee | Moorfields NHS FT |
| Sarah | Martin | Moorfields NHS FT |
| Mary | Masih | Moorfields NHS FT |
| Dhakshayini | Muhundhakumar | Moorfields NHS FT |
| Steve | Napier | Moorfields NHS FT |
| Luke | Nicholson | Moorfields NHS FT |
| Natalie | O'Shea | Moorfields NHS FT |
| Ella | Preston | Moorfields NHS FT |
| Kimberley | Quan | Moorfields NHS FT |
| Tulga | Reis | Moorfields NHS FT |
| Nick | Roberts | Moorfields NHS FT |
| Ana | Sanchez | Moorfields NHS FT |
| Kathryn | Scotcher | Moorfields NHS FT |
| Sobha | Sivaprasad | Moorfields NHS FT |
| Jon | Spencer | Moorfields NHS FT |
| Peter | Thomas | Moorfields NHS FT |
| Rachel | Thompson | Moorfields NHS FT |
| Karen | Titmus | Moorfields NHS FT |
| Jonathan | Wilson | Moorfields NHS FT |
| Nick | Hynes | Somo Global |
| Tom | Blair | Ubisense |
| Paul | Webster | Ubisense |
| Omar | Abolnaga | UCL |
| Farbod | Afshar Bakeshloo | UCL |
| Kenan | Arifoglu | UCL |
| Nick | Burt | UCL |
| Caroline | Clarke | UCL |
| Dolores | Conroy | UCL |
| Ian | Eames | UCL |
| Ecem | Ergin | UCL |
| Iqbal | Fahmi | UCL |
| Paul | Foster | UCL |
| Susana | Frazao Pinheiro | UCL |
| Naomi | Fulop | UCL |
| Saheli | Gandhi | UCL |
| Steven | Gray | UCL |
| Xiaojia | Guo | UCL |
| Rouba | Ibrahim | UCL |
| Hanna | James | UCL |
| Melih | Kamaoglu | UCL |
| Stephanie | Kumpunen | UCL |
| Xiaoming | Li | UCL |
| Josefine | Magnusson | UCL |
| Dominika | Matusiak | UCL |
| Grant | Mills | UCL |
| Siyabonga | Ndwandwe | UCL |
| Pei Li | Ng | UCL |
| Giovanni | Ometto | UCL |
| Rosica | Pachilova | UCL |
| Angus | Ramsay | UCL |
| Yaman | Rawas Kalaji | UCL |
| Daniel | Rennie | UCL |
| Irinie | Roufaeel | UCL |
| Kerstin | Sailer | UCL |
| Mine | Sak Acur | UCL |
| Peter | Scully | UCL |
| Lina | Song | UCL |
| Anne | Symons | UCL |
| Yue | Tang | UCL |
| Ahmed | Tarek Zaky Fouad | UCL |
| Melike | Toprak | UCL |
| Jemima | Unwin Teji | UCL |
| Martin | Utley | UCL |
| Duncan | Wilson | UCL |
| Dongyuan | Zhan | UCL |
| Jocelyn | Cammack | UCL and Moorfields NHS FT |
| Paula | Lorgelly | University of Auckland |
