# Supplementary material for: Impact of a community-based asynchronous review clinic on appointment attendance delays across an eye hospital network in London, UK: an interrupted time series analysis
Source: BMJ Open. 2025 Jul 15;15(7):e098820. doi: 10.1136/bmjopen-2025-098820 (PMC12273167; doi:10.1136/bmjopen-2025-098820)
Supplement: Supplementary file 2 [file bmjopen-2025-098820.reviewer_comments.pdf]

PEER REVIEW HISTORY

BMJ Open publishes all reviews undertaken for accepted manuscripts. Reviewers are asked to complete a checklist review form and are provided with free text boxes to elaborate on their assessment. These free text comments are reproduced below.

ARTICLE DETAILS

Title (Provisional)

Impact of a Community-Based Asynchronous-Review Clinic on Appointment Attendance Delays Across an Eye Hospital Network in London, UK: An Interrupted Time Series Analysis

Authors

Ndwandwe, Siyabonga; Fu, Dun Jack; Adesanya, Joy; Bazo-Alvarez, Juan Carlos; Ramsay, Angus I. G.; Fulop, Naomi J; Magnusson, Josefine; Napier, Steve; Cammack, Jocelyn; Baker, Helen; Kumpunen, Stephanie; Alarcón Garavito, Germán Andrés; Elphinstone, Holly; Mills, Grant; Scully, Peter; Symons, Anne; Webster, Paul; Wilson, Jonathan; Khaw, Peng Tee; Sivaprasad, Sobha; Jayaram, Hari; Foster, Paul J; Clarke, Caroline S

VERSION 1 - REVIEW

**Reviewer**	**1**
**Name**	**Sanjay, Srinivasan**
**Affiliation**	**Narayana Nethralaya**
**Date**	**31-Mar-2025**
**COI**	**None**

Impact of a Community-Based Asynchronous-Review Clinic on Appointment Attendance Delays Across an Eye Hospital Network in London, UK – Project HERCULES

Abstract

Objective- First sentence is too long, can split the sentence

COVID-19 expand before abbreviation

Percentages should be followed by n=

Deprived half of the population was defined as

COVID national lockdown period should be stated

Article

1. NHS is under immense strain- Is it the same reference 1?

2. Again the 2nd sentence is too long

3. Ophthalmology the busiest specialty- reference

4. Ophthalmic technicians- intensive training for how long and what were their qualifications before training?

5. Page 19 line 60- insert figure 1 means?

6. How do you define stable glaucoma and macular degeneration patients in your study?

7. P24 line 155 remove (

8. Line 157/158, please provide n=

9. Line 160 (39%, percentage is in brackets which is not uniform with the rest of the text

10. Model 1 In Error! Reference source not found.).- what does this mean? There are also 2 full stops

11. P33 line 232 n=

**Reviewer**	**2**
**Name**	**Anstice, Nicola S.**
**Affiliation**	**Flinders University South Australia, Optoimetry and Vision Science**
**Date**	**06-Apr-2025**
**COI**	**None**

This is a very well written manuscript which evaluates changes in appointment waiting times as a result of the introduction of a novel model of asynchronous telehealth during the COVID-19 panademic. The authors have used extensive and robust modelling to evaluate this telehealth model, which is adequately supported through the supplementary materials provided. However, before publication I have a few minor concerns that could be considered:

Introduction Lines 35-36 - for non-clinicians it may be worth pointing out that the medical retina category including patients living with diabetes and AMD

Methods Lines 58-60 - I think a reference has got lost here and that figure 1 is not mean to be inserted at this point. Could the authors check this section of the manuscript for accuracy?

Methods Line 64 - what concerns did the authors have about the 'data quality issues' for patients >100 years of age?

Methods (general comment) - there is no information provided about the protocols undertaken as part of the intervention site clinics. What measures were undertaken by the ophthalmic technician and how were these reviewed? What happened to patients that had previously stable conditions that had now progressed? What referral pathways were available for these patients?

Methods Line 123 - consider removing 'please' before 'see supplementary material...'

Methods Line 144 - what does the acronym BRC stand for?

Results Line 155-157 - again I think something has happened with the formatting here as Figure 1 has been included earlier in the manuscript

Results Table 1 - did any of the patients identify as non-binary in the data that was collected? Was this an option that the patients could select as part of their health records?

Results Line 170 - 'Error! Reference source not found' message should be replaced with the appropriate reference

Discussion - Limitations of the study - one of the limitations of the study is that it only reports on the delay to attendance (and the decreases in this delay that were seen once the intervention site clinic was established). However, there are other important indicators of the efficacy of such an interventions such as the accuracy of findings and the adherence to the protocols developed. While these may be outside the scope of this manuscript, it would be good to acknowledge that these are important factors to also consider.

VERSION 1 - AUTHOR RESPONSE

Reviewer 1:<br><br>Objective- First sentence is too long, can split the sentence	We thank the reviewer for this helpful suggestion. We have split this sentence and it reads much better now.
COVID-19 expand before abbreviation	This has been addressed in the last paragraph of the introduction.
Percentages should be followed by n=	Thank you for highlighting this. We have addressed the issue wherever possible; however, this was not always feasible when citing statistics from published reports. Additionally, we have removed a sentence following email correspondence with The Royal College of Ophthalmologists (policy@rcophth.ac.uk / jordan.marshall@rcophth.ac.uk) regarding their 2022 workforce census. The original statement read:<br><br>"In 2022, 76% of NHS ophthalmology units did not have enough consultants to meet patient demand, whilst 52% found it increasingly difficult to recruit consultants [2]."<br><br>Our correspondence clarified that the census was based on 55 responses, and the figure of 76% likely referred to 42 respondents answering "No" to whether their unit had sufficient consultants. However, neither the report nor the correspondence confirmed whether each response represented a unique NHS ophthalmology unit. As a result, we have removed this sentence from the introduction.

	We have also modified a paragraph in the discussion section to highlight the denominator. We state that:<br><br>"data from Specsavers' Hindsight Report (2021), which conducts 46% of eye checks in the UK, estimated that 4.3 million eye tests were not delivered in 2020 in the UK compared to 2019, representing a 23% decline [25]. Using NHS Digital's Hospital Episode Statistics (HES), the report further highlighted decrease in ophthalmology treatments: outpatient attendances and day case procedures declined by 36% and 45% respectively between March and December 2020 compared to the same period in 2019[25]"<br><br>The report itself does not state the corresponding figures for ophthalmology outpatient attendance and day case procedures. Nonetheless, these figures are reported at the national level, and we believe the reported capture the scale of the problem.
Deprived half of the population was defined as	Thanks for suggesting this clarification. This has been addressed in the results section (line 157) as below:<br><br>• "There was an even split between males and females (51%, n=35,048) and 49%, n=34,204) and patients older than 65 years accounted for 54% (n=37,824) of attendances whilst those in the more deprived half of the population (IMD decile 1 to 5) accounted for 53% (n=37,014) of attendances"
COVID national lockdown period should be stated	This has been stated in the abstract as mid-March 2020. We further reiterate the lockdown timelines as 'spring 2020 COVID-19 national lockdown and specifically state that the first COVID-19 national lockdown in the week of 16 March 2020, with the caveat that whilst the UK officially began the first lockdown on 23 March 2020, some changes in people's behaviour preceded the official lockdown declaration and cite the Greater London Authority COVID-19 mobility report and a report from the Institute for Government [17,18]

NHS is under immense strain- Is it the same reference 1?	Yes, we thank the reviewer for pointing this out. It is a general statement and we are also using reference [1] to support it. We have clarified this in the manuscript.
Again the 2nd sentence is too long	Thanks for this comment. We have reworded to improve the sentence structure.
Ophthalmology the busiest specialty- reference	We have revised this sentence and cited NHS Digital's *Hospital Outpatient Activity 2022-23* report [2].
Ophthalmic technicians- intensive training for how long and what were their qualifications before training?	Thank you for this clarification. Technicians had not worked in health care before. They had GCSE English and Maths, or equivalent customer service/public facing experience. Their training was 6-12 weeks and was determined by achieving specific task related competencies. A buddy system was used, whereby each new staff member was paired with a more experienced colleague. Qualitative work from our research group, in preparation for publication, highlights that the staff greatly appreciated this intensive and personalised training system. There was an examination at 6 months from initiation to verify knowledge around newly acquired skills.<br><br>We have now revised the introduction to highlight this information.
Page 19 line 60- insert figure 1 means?	This statement highlights where figure 1 ought to be inserted. We have modified the statement to "Please Insert Figure 1 here" for clarity, and note that the figures themselves are uploaded to the journal system in separate files as required.
How do you define stable glaucoma and	Thank you for this comment. We have clarified where we had stated the definition of stable patients (line 68). It now reads as follows:

macular degeneration patients in your study?	“We further excluded non-stable patients. For purposes of this work, we defined stable patients as those with an LCAD longer than 6 months from their previous attendance or those not seen more than four weeks sooner than the stated LCAD. The former would indicate that the patient’s condition was not stable, whilst the latter would suggest an urgent visit might have occurred, i.e. the attendance would not have been the scheduled regular monitoring visit.”
P24 line 155 remove (	Thank you for this suggestion, we have fixed this in the manuscript by removing the mid-sentence reference to the table and adding it at the end of the sentence.
Line 157/158, please provide n=	Thank you for this suggestion, we have fixed this in the manuscript.
Line 160 (39%, percentage is in brackets which is not uniform with the rest of the text	Thank you. We have revised this sentence from:<br>“Furthermore, the main facility site in central London accounted for the highest proportion of attendances (39%, n = 27,339), whilst the intervention site and all other sites accounted for 5% (n= 3,137) and 56% (n= 38,781) respectively.”<br>To:<br>“Furthermore, the main facility site in central London accounted for 39% (n = 27,339) of attendances, whilst the intervention site and all other sites accounted for 5% (n= 3,137) and 56% (n= 38,781) respectively.”
Model 1 In Error! Reference source not found.).- what does this mean? There are also 2 full stops	This must have been a cross-referencing error. We have fixed this now and thank the reviewer for spotting it.

<table>
<tr><td>P33 line 232 n=</td><td>Thank you for picking this up. Whilst the reports referenced in this section did not consistently state the corresponding figures, we have modified it to provide clarity and context to better represent the statistic. It now reads as follows:

“For example, data from Specsavers' Hindsight Report (2021), which conducts 46% of eye checks in the UK, estimated that <u>4.3 million eye tests</u> were not delivered in 2020 in the UK compared to 2019, representing a 23% decline[25]. Using NHS Digital’s Hospital Episode Statistics (HES), the report further highlighted a decrease in ophthalmology treatments: outpatient attendances and day case procedures declined by 36% and 45% respectively between March and December 2020 compared to the same period in 2019[25]”

The figures corresponding to outpatient attendances and day case procedures were not stated; however, since these are reported at the national level, we believe that the proportions give a picture of the scale of the problem at hand.</td></tr>
<tr><td>Review 2</td><td></td></tr>
<tr><td>Introduction Lines 35-36 - for non-clinicians it may be worth pointing out that the medical retina category including patients living with diabetes and AMD</td><td>Thanks for this helpful comment. To improve clarity, we have now revised this sentence from:

“Initially, services were for monitoring patients with stable glaucoma, macular degeneration, and diabetic retinopathy (henceforth categorised as glaucoma and medical retina).”

To:

“Initially, services were for monitoring patients with stable glaucoma, age-related macular degeneration, and diabetic retinopathy (henceforth broadly categorised as glaucoma and medical retina).”</td></tr>
<tr><td>Methods Lines 58-60 - I think a reference has got lost here and that figure 1 is not mean to be inserted at this point. Could the</td><td>Yes, thanks very much, reference to figure 1 (data cleaning process) was inappropriate in this section. We have now moved this sentence, it is instead now the opening sentence in the results section and reads as follows:</td></tr>
</table>

authors check this section of the manuscript for accuracy?	"Having followed the data cleaning process highlighted in **Error! Reference source not found.**, our final and analysed sample had 69,257 attendances, from 39,357 patients, across 13 sites within the eye care facility network."
Methods Line 64 - what concerns did the authors have about the 'data quality issues' for patients >100 years of age?	For the population we are analysing, we did not anticipate patients older than 100 years, particularly at the first recorded appointment in a series within the data. Our largest concern was that this might be due to data entry error, for example entering the date of birth as 1914 instead of 1941.
Methods (general comment) - there is no information provided about the protocols undertaken as part of the intervention site clinics. What measures were undertaken by the ophthalmic technician and how were these reviewed? What happened to patients that had previously stable conditions that had now progressed? What referral pathways were available for these patients?	Thank you for highlighting this, we have added the key details in the introduction. It now reads as follows:<br>"Tests at the intervention site were carried according to eye care facility network's standard operating procedures and differed between disease categories, per guidelines. Test scans were uploaded to the hospital network's secure online system and later reviewed remotely by specialist clinicians, who determined whether to discharge the patient, continue follow-ups through the asynchronous/virtual pathway, or escalate to a face-to-face follow-up."<br><br>To detail the above (although we have not included all this detail in the updated manuscript as we felt it might be too much, but we can add in more of this to the manuscript if preferred), we wish to state that the examination carried out at the intervention site differed between disease categories of patient. These were carried according to Moorfields Eye Hospital standard operating procedures. For all patients, there was an assessment of habitual and best corrected visual acuity using a Thompson Snelling chart. This was followed by an assessment of pinhole acuity, if best correct visual acuity was below 6/6. Staff carried out a systematic review of general and ophthalmic health, together with a

	review of medication usage and any potential side-effects, any new health events, any new drug allergies, and any salient information regarding relevant symptoms.<br><br>For glaucoma patients, auto refraction was carried out. Intraocular pressure was measured using a Reichert Ocular Response Analyser (ORA). This gave measures of corneal corrected intraocular pressure, corneal hysteresis, Goldmann calibrated intraocular pressure and a waveform score, indicating quality of the measure. Visual field testing on a Zeiss Humphrey automated perimeter, white on white, 24–2 test pattern running a SITA fastest testing protocol. An assessment of the peripapillary retinal nerve layer thickness and macular ganglion cell layer thickness was performed with a Zeiss Cirrus optical coherence tomographer and finally an ultra-widefield colour fundus image was captured with an Optos California machine.<br><br>For medical retina patients had a measurement of interocular pressure using a handheld iCare tonometer. Pupils were dilated with two drops of tropicamide 1% and two drops of phenylephrine 2.5%, each administered 10 minutes apart. Once pupil dilation had been achieved, patients underwent macular OCT on a Heidelberg Spectralis machine and an ultra-widefield retinal image was captured with an Optos California machine.<br><br>The data from these examinations was reviewed by specialist Moorfields clinicians, and a decision made to discharge, continue follow up in virtual clinic pathway, or to escalate to face to face review.
Methods Line 123 - consider removing	We have revised the statement to “see supplemental material” to ensure consistency with the rest of the

'please' before 'see supplementary material...'	manuscript where readers are directed to the supplemental materials for further details.
Methods Line 144 - what does the acronym BRC stand for?	We have now amended this sentence and have Biomedical Research Council (BRC) fully spelled out before to give the abbreviation.
Results Line 155-157 - again I think something has happened with the formatting here as Figure 1 has been included earlier in the manuscript	Thank you for spotting this. We have revised this by removing any reference to figure 1 in the methods section and added it to the results section. Also see previous response to similar comment above.
Results Table 1 - did any of the patients identify as non-binary in the data that was collected? Was this an option that the patients could select as part of their health records?	Thank you for highlighting this. Unfortunately, this information was not collected, as the health records at the time only allowed three options for this variable: male, female, or no response/left blank.
Results Line 170 - 'Error! Reference source not found' message should be replaced with the appropriate reference	This must have been a cross-reference error that manifested during file conversion. We have made these static in the final submitted version to avoid this issue appearing again.
Discussion - Limitations of the study - one of the limitations of the study is that it only reports on the	We thank the reviewer for their thoughtful consideration of our work and their insightful suggestion. We agree that these are important aspects that should ideally form part of a larger or future analysis, although the reviewer correctly mentions that they are not within the scope of this work. We

delay to attendance (and the decreases in this delay that were seen once the intervention site clinic was established). However, there are other important indicators of the efficacy of such an interventions such as the accuracy of findings and the adherence to the protocols developed. While these may be outside the scope of this manuscript, it would be good to acknowledge that these are important factors to also consider.	have added in some discussion of this in section 4.2 in the manuscript.<br><br>In case it is of interest to the reviewers (although we have not included all the below detail in the updated manuscript as we felt it might be too much, but we can add in more of this to the manuscript if preferred), we provide the following information:<br><br>With regards to "accuracy of findings", we wonder if the reviewer is thinking of reproducibility of clinical decision making. We have previously studied and reported this. See:<br><br>Clarke J, Puertas R, Kotecha A, Foster PJ, Barton K. Virtual clinics in glaucoma care: face-to-face versus remote decision-making. British Journal of Ophthalmology. 2017 Jul 1;101(7):892-5. DOI: 10.1136/bjophthalmol-2016-308993<br><br>Where we concluded that the low rate of adverse misclassification, the typically slow progression of most glaucoma cases, and regular patient reassessment together support virtual clinics as a safe and logistically feasible option for selected glaucoma patients. We had highlighted these findings in the introduction (lines 45 – 53)<br><br>With regards to "adherence to the protocols developed", this was ensured through Moorfields standard operating procedures and the use of an electronic medical record (EMR) system, which mandates completion of key review and clinical decision tasks before a workstream can progress. All clinician activity within the EMR is recorded.
